# Molecular mechanisms of Huanglian Jiedu decoction in treating Alzheimer’s disease by regulating microbiome *via* network pharmacology and molecular docking analysis

**DOI:** 10.3389/fcimb.2023.1140945

**Published:** 2023-03-16

**Authors:** Renyuan Zheng, Shenggan Shi, Qin Zhang, Shuqin Yuan, Tong Guo, Jinlin Guo, Peidu Jiang

**Affiliations:** ^1^ Sichuan Key Laboratory of Noncoding RNA and Drugs, School of Basic Medical Sciences, Chengdu Medical College, Chengdu, China; ^2^ Personalized Drug Therapy Key Laboratory of Sichuan Province, Department of Pharmacy, Sichuan Provincial People’s Hospital, University of Electronic Science and Technology of China, Chengdu, China; ^3^ State Key Laboratory of Southwestern Chinese Medicine Resources, College of Pharmacy, Chengdu University of Traditional Chinese Medicine, Chengdu, China; ^4^ Chongqing Key Laboratory of Sichuan-Chongqing Co-construction for Diagnosis and Treatment of Infectious Diseases Integrated Traditional Chinese and Western Medicine, College of Medical Technology, Chengdu University of Traditional Chinese Medicine, Chengdu, China

**Keywords:** Huanglian Jiedu decoction, Alzheimer’s disease, network pharmacology, microbial flora, molecular docking

## Abstract

**Background:**

Huanglian Jiedu decoction (HLJDD) is a famous traditional Chinese medicine prescription, which is widely used in the treatment of Alzheimer’s disease (AD). However, the interaction between bioactive substances in HLJDD and AD-related targets has not been well elucidated.

**Aim:**

A network pharmacology-based approach combined with molecular docking was performed to determine the bioactives, key targets, and potential pharmacological mechanism of HLJDD against AD, through the regulation of microbial flora.

**Materials and methods:**

Bioactives and potential targets of HLJDD, as well as AD-related targets, were retrieved from Traditional Chinese Medicine Systems Pharmacology Analysis Database (TCMSP). Key bioactive components, potential targets, and signaling pathways were obtained through bioinformatics analysis, including protein-protein interaction (PPI), Gene Ontology (GO), and Kyoto Encyclopedia of Genes and Genomes (KEGG) analysis. Subsequently, molecular docking was performed to predict the binding of active compounds with core targets.

**Results:**

102 bioactive ingredients of HLJDD and 76 HLJDD-AD-related targets were screened. Bioinformatics analysis revealed that kaempferol, wogonin, beta-sitosterol, baicalein, acacetin, isocorypalmine, (S)-canadine, (R)-canadine may be potential candidate agents. AKT1, TNF, TP53, VEGFA, FOS, PTGS2, MMP9 and CASP3 could become potential therapeutic targets. 15 important signaling pathways including the cancer pathway, VEGF signaling pathway, and NF-κB signaling pathway might play an important role in HLJDD against AD. Moreover, molecular docking analysis suggested that kaempferol, wogonin, beta-sitosterol, baicalein, acacetin, isocorypalmine, (S)-canadine, and (R)-canadine combined well with AKT1, TNF, TP53, VEGFA, FOS, PTGS2, MMP9, CASP3, respectively.

**Conclusion:**

Our results comprehensively illustrated the bioactives, potential targets, and possible molecular mechanisms of HLJDD against AD. HLJDD may regulate the microbiota flora homeostasis to treat AD through multiple targets and multiple pathways. It also provided a promising strategy for the use of traditional Chinese medicine in treating human diseases.

## 1 Introduction

Alzheimer’s disease (AD), also known as senile dementia, is a degenerative disease of the central nervous system characterized by progressive cognitive impairment and memory loss (Soria Lopez et al., 2019). The histopathological manifestations of AD are brain atrophy, amyloid β (Aβ) protein deposition, neuronal loss, senile plaques, neurofibrillary tangles, etc. (Loera-Valencia et al., 2019; Breijyeh and Karaman, 2020). In addition to these definite pathological injuries, the brain’s immune response is involved in the course of AD (Henstridge et al., 2019). Although the pathological mechanisms underlying AD remain controversial, Aβ peptide is believed to be the central participant. Recently, Opare (Opare and Rauk, 2019) and Samanta (Samanta et al., 2019) demonstrated that Aβ-related peptides were the initiators of AD, and the imbalance between Aβ accumulation and clearance was the main cause of AD. The drugs currently approved for clinical treatment of AD are based on neuroprotective agents (e.g., neurotransmitter generators or neurotransmitter receptor agonists/antagonists). For example, donepezil is a centrally reversible acetylcholinesterase (AChE) inhibitor that increases ACh levels and improves cognitive function in patients with AD (Ma et al., 2018). At present, more than 1000 drugs have been developed to treat AD worldwide, but only 6 drugs have been approved for clinical use by FDA. Moreover, these drugs are usually acetylcholinesterase inhibitors, which mainly target a single molecule and can only play a partial role in slowing down the progress of AD. They cannot reverse the clinical process of the disease, and have no obvious therapeutic effect on AD related to the pathogenesis of multiple targets and multiple pathways. GV-971 is a low-molecular-weight acidic oligosaccharide compound extracted from marine brown algae. As the effect of reversing the cognitive impairment of AD, it was approved by FDA in the third clinical phase of AD in 2020. Its mechanism of action is mainly to inhibit gut dysbiosis and reverse the neuroinflammation and cognitive impairment of AD patients through the microbiota-gut-brain axis. The success of GV-971 in reversing and treating AD brings a new idea for drug development of multi-target intervention in AD. Therefore, the mechanism of traditional Chinese medicine (TCM) in treating AD, which is characterized by multi-component and multi-target effects, needs to be further studied. TCM has a long clinical application in China and is an important tool for the treatment of many complex diseases with multiple targets, such as AD (Pei et al., 2020) and cardiovascular diseases (Hao et al., 2017). Huanglian Jiedu decoction (HLJDD) is a classic prescription with heat-clearing and detoxifying effects, mainly by *Rhizoma coptidis* (*Ranunculaceae*), *Radix scutellariae* (*Labiatae*), *Cortex phellodendri* (*Rutaceae*) and *Fructus gardeniae* (*Rubiaceae*) in a ratio of 3:2:2:3 mixed. HLJDD is widely used in China and Japan to treat cerebral ischemia and to exert neuroprotective effects (Zhang et al., 2014). Sun et al. found four target proteins related to AD and two pathways related to neuroinflammation through network pharmacology, and speculated that HLJDD may exert its anti-AD effect by scavenging/reducing Aβ in the brain and inhibiting hyperphosphorylation of tau protein through insulin signaling pathway (Sun et al., 2017). However, the relationship between HLJDD and microbial flora (gut microbiota, oral microorganisms, skin microorganisms, etc.) *in vivo* needs further study. Whether HLJDD exerts its therapeutic effects on AD through regulating microbial flora homeostasis is still unclear.

**Figure 1:**
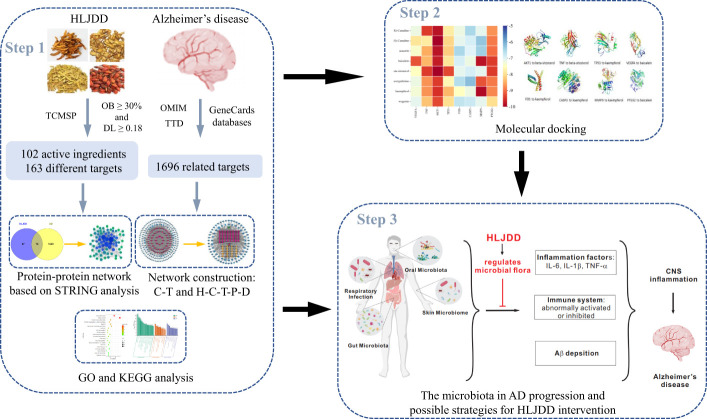
The summary and description of the study workflow in the potential mechanisms of HLJDD in treating AD. Network pharmacology was used to analyze the crucial ingredients and key targets of HLJDD in the treatment of AD; molecular docking revealed that eight candidate compounds could bind well with eight candidate targets, respectively. It is speculated that HLJDD exerts its anti-AD effect by regulating the homeostasis of microbial flora *in vivo* and inhibiting neuroinflammation.

Network pharmacology and molecular docking analysis was performed in this study to explore the possible mechanisms of HLJDD in the treatment of AD through constructing the network of the “herb-component-target-pathway-disease”. It will provide a new theoretical support for the clinical treatment of AD and a reference for screening bioactive components with potential medicinal value. The workflow of the whole process of our study was shown in **Figure 1**.

## 2 Materials and methods

### 2.1 Screening and target prediction of active components of HLJDD

The active components of *Rhizoma coptidis*, *Radix scutellariae*, *Cortex phellodendri* and *Fructus gardeniae* in HLJDD were searched by application analysis platform and database system pharmacology of Chinese medicine (TCMSP, https://tcmspw.com/tcmsp.php) (Ru et al., 2014) and the targets of active components were predicted. We screened the active ingredients that meet both oral bioavailability (OB) ≥ 30% and drug-like (DL) ≥ 0.18 by pharmacodynamics (Wang and Wang, 2017; Wang et al., 2019a).

### 2.2 Prediction of drug and disease related targets

TCMSP was used to predict the target of active ingredients. The obtained drug components were searched in the TCMSP and BATMAN-TCM database platforms to obtain the target proteins corresponding to each drug component, and the protein targets were compared in the Uniprot protein database (https://www.uniprot.org/) (The UniProt Consortium, 2017) as standard gene names, and non-human genes were removed. GeneCards (https://www.genecards.org/) (Safran et al., 2010), OMIM (https://omim.org/search/advanced/) (Hamosh et al., 2005), TTD (https://db.idrblab.net/ttd/) (Zhou et al., 2022) and other databases were searched with “Alzheimer disease” as the key word. The gene targets searched in GeneCards database were deleted according to Relevance score ≥ 1. The target genes obtained from these three databases were combined, and the disease genes related to AD were obtained after deleting the duplicates.

### 2.3 Screening of common targets of diseases and drugs and construction of PPI network

In order to clarify the mechanism of action of drug targets and disease targets at the protein level, drug genes and disease genes were submitted to the online Venny 2.1 mapping platform (https://bioinfogp.cnb.csic.es/tools/venny/index.html) to draw Venn diagram and obtain the intersection genes of drug and component targets. The intersection genes were imported to STRING gene database (https://string-db.org/cgi/input) (Szklarczyk et al., 2019) to construct protein interaction network model. The species was set as “Homo sapiens”, the minimum interaction threshold was set as “medium confidence”, and the free protein was hidden to obtain the PPI network. The PPI network download was saved in Tsv format and imported into Cytoscape 3.9.1 software for visualization (Shannon et al., 2003).

### 2.4 GO and KEGG enrichment analysis

For the screened core targets, the DAVID platform website (https://david.ncifcrf.gov) was used for Gene Ontology (GO) functional annotations and Kyoto Encyclopedia of Genes and Genomes (KEGG) pathway enrichment analysis, and *P*-value was set to screen the top 10 important pathways.

### 2.5 Constructing the “composition-target” network and “herb-component-target-pathway-disease” network

Cytoscape 3.9.1 software was used to construct the composition-target network and the herb-component-target-pathway-disease (H-C-T-P-D) interaction network. Node represents active components, drugs, diseases and targets, while edge represents the relationship between different nodes. Through the Network Analyzer function of Cytoscape software, combined with the main active ingredients, core targets and concentrated main signaling pathways, the possible core active ingredients of HLJDD in the treatment of AD were speculated. It can also show the functional relationship of traditional Chinese medicine, active ingredients, targets and pathways in the treatment of AD.

### 2.6 Validation molecular docking

The 3D structures of the main active ingredients in HLJDD were downloaded from the TCMSP database while the 3D structure of the core target protein (top 8 of degree in PPI network) treated by HLJDD was downloaded from the Worldwide Protein Data Bank (PDB) database (https://www.rcsb.org/). They were imported into PyMOL and AutoDockTools and Autodock vina (Trott and Olson, 2010) software to remove water molecules and small molecule ligands, hydrogenation, and then molecular docking of receptors and ligands was performed. The binding ability and stability of the targets and active ingredients were evaluated by docking score and hydrogen bond number. The docking results were visualized using PyMOL software.

## 3 Results

### 3.1 The active components and effective targets of HLJDD

Based on TCMSP database, 102 different active ingredients of HLJDD were screened, including 14 in *Coptis chinensis*, 36 in *Scutellaria baicalensis*, 37 in *Phellodendri chinensis* and 15 in *Gardenia jasminoides*. Cytoscape 3.9.1 software was used to screen the active ingredients with OB≥30% and DL≥0.18 in HLJDD, as shown in **Table 1**.

**Table 1:** Chemical information sheet of major active ingredients.

Mol ID	Molecule name	OB%	DL	Degree	Herb
MOL000422	Kaempferol	41.88	0.24	37	Zhizi
MOL000173	wogonin	30.68	0.23	23	Huangqin
MOL000358	beta-sitosterol	36.91	0.75	21	Huangqin, Huangbo, Zhizi
MOL002714	baicalein	33.52	0.21	21	Huangqin
MOL001689	acacetin	34.97	0.24	18	Huangqin
MOL000790	Isocorypalmine	35.77	0.59	17	Huangbo
MOL001455	(S)-Canadine	53.83	0.77	16	Huangbo
MOL002903	(R)-Canadine	55.37	0.77	15	Huanglian

OB, oral bioavailability; DL, drug-likeness.

1004 different drug targets were screened using the TCMSP and BATMAN-TCM database which included 148 targets in *Coptis chinensis*, 498 targets in *Scutellaria baicalensis*, 218 targets in *Phellodendron amurense*, and 140 targets in *Gardenia jasminoides Ellis*. After merging and deleting the repeated values, 163 targets were obtained, and the targets information were standardized by Uniprot database.

### 3.2 Related targets for disease

After combining the OMIM, TTD, and GeneCards databases and deleting repeated targets, 1696 AD-related targets were finally obtained.

### 3.3 Common targets for diseases and drugs

**Figure 2:**
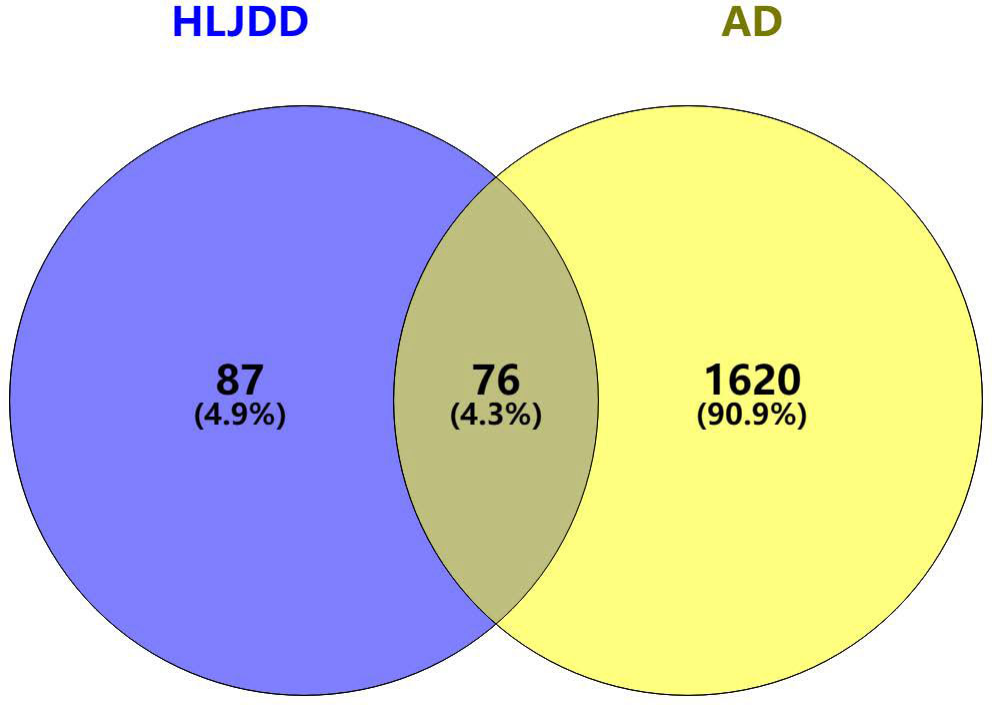
Disease-drug target Venn diagram.

1696 AD-related targets and 163 HLJDD drug predicted targets were imported using the Venny online mapping platform. After mapping, 76 intersection targets of HLJDD and AD were obtained (**Figure 2**).

### 3.4 Construction and analysis of HLJDD-AD-related PPI network

**Figure 3:**
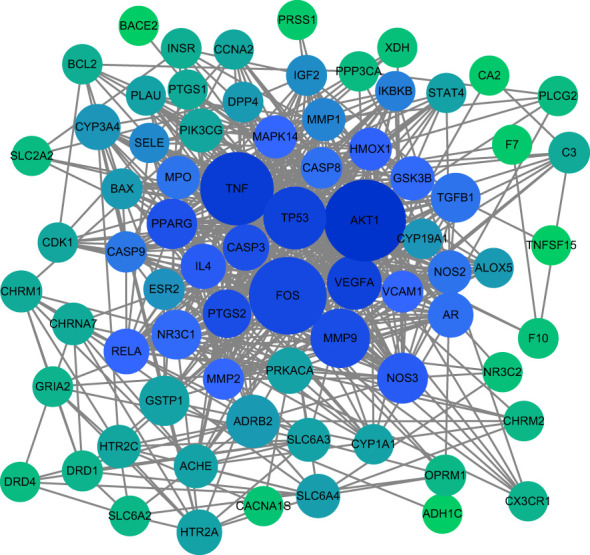
Core target PPI network. As shown in the figure, the darker color of the circle is proportional to its importance in this network.

76 intersection targets were then imported into the STRING platform to construct a PPI network. 74 nodes and 585 edges were obtained using this platform. Node size and color indicated the size of the value. The double median of “Degree” that is, “Degree ≥30” was used to screen the intersection targets. Thus, 8 target genes with the highest degree of AD treated by HLJDD were shown in **Table 2**. The PPI network information obtained from the STRING11.5 database was imported into Cytoscape 3.9.1 software for visualization (**Figure 3**).

**Table 2:** Core target information table.

Target	Degree	Betweenness centrality (BC)	Closeness centrality (CC)
AKT1	49	0.124526	0.744898
TNF	45	0.099243	0.708738
TP53	42	0.067676	0.682243
VEGFA	42	0.04322	0.682243
FOS	39	0.111585	0.675926
PTGS2	37	0.024935	0.651786
MMP9	37	0.057281	0.640351
CASP3	36	0.018634	0.640351

### 3.5 GO and KEGG enrichment analysis

**Figure 4:**
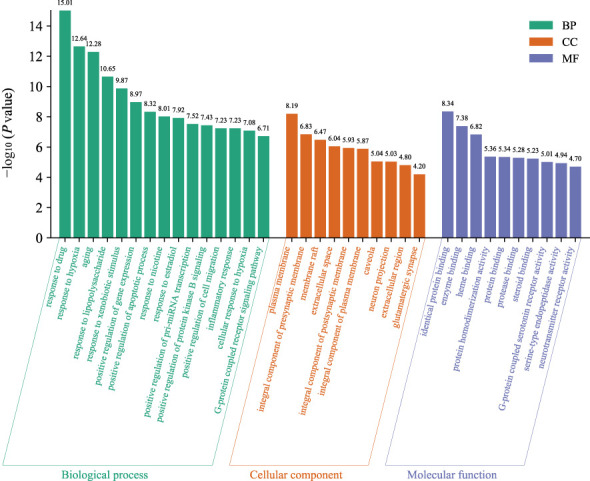
The GO function analyzes the histogram. BP is marked in teal, CC in sienna, and MF in steel blue. The bar graph is obtained by Bioinformatics Platform.

The GO function enrichment analysis of the 76 core targets was performed on the Metascape platform, and 943 GO items were obtained, including 419 “Biological Processes (BP)”, 54 “Cellular Components (CC)”, and 88 “Molecular Functions (MF) “. The first 15 “Biological Processes” items, 10 “Cellular Components” items, and 10 “Molecular Functions” items were selected based on the *P* value for visual analysis (**Figure 4**). Results showed that the treatment of AD by HLJDD mainly involved BP such as aging, response to drug, response to hypoxia, response to lipopolysaccharide, response to xenobiotic stimulus, response to nicotine, response to estradiol, positive regulation of pri-miRNA transcription, positive regulation of gene expression, positive regulation of apoptotic process, etc. These targets passed through identical protein binding, enzyme binding, heme binding, protein homodimerization activity, protein binding, protease binding, steroid binding, G-protein coupled serotonin receptor activity, serine-type endopeptidase activity, neurotransmitter receptor activity and other functions, and they played a role in the plasma membrane, presynaptic membrane, membrane raft, extracellular space, postsynaptic membrane, caveola, neuron projection, extracellular region, glutamatergic synapse, etc.

**Figure 5:**
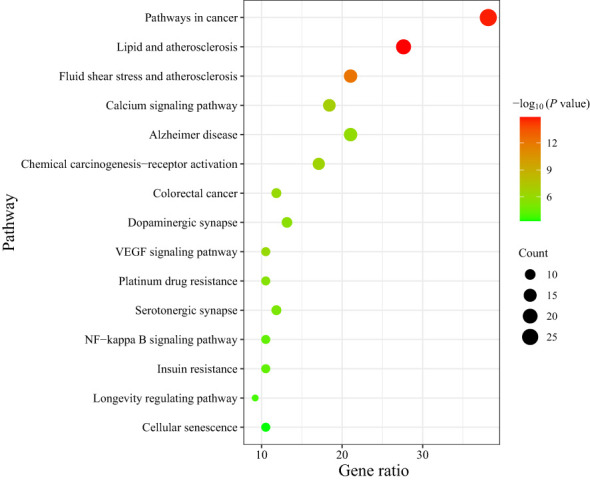
KEGG enrichment bubble diagram of the treatment of AD by HLJDD.

137 signal pathways were enriched by KEGG pathway analysis of the core targets using the DAVID platform. According to the *P* value and the number of genes, 15 signal pathways with high probability were screened out for visual analysis as shown in **Table 3** and **Figure 5** (bubble diagram was plotted by https://www.bioinformatics.com.cn). Moreover, **Figure 5** showed that HLJDD treatment of AD may be mainly related to calcium, VEGF, and NF-κB signaling pathway, lipid and atherosclerosis, chemical carcinogenesis-receptor activation, dopaminergic synapse, platinum drug resistance, etc.

**Table 3:** KEGG pathway enrichment results.

Term	%	Count	P value	Related genes
Hsa05200: Pathways in cancer	38.15789	29	2.28E-15	*GSK3B, GSTP1, PTGS2, RELA, CASP9, IKBKB, CASP8, CASP3, PLCG2, STAT4, AKT1, HMOX1, PRKACA, TGFB1, NOS2, MMP1, MMP2, IGF2, FOS, MMP9, ESR2, VEGFA, IL4, CCNA2, AR, BCL2, BAX, PPARG, TP53*
Hsa05417: Lipid and atherosclerosis	27.63158	21	1.37E-15	*GSK3B, VCAM1, NOS3, MMP1, FOS, MAPK14, SELE, TNF, MMP9, RELA, CASP9, IKBKB, PPP3CA, CASP8, CASP3, CYP1A1, BCL2, BAX, AKT1, PPARG, TP53*
Hsa05418: Fluid shear stress and atherosclerosis	21.05263	16	8.56E-13	*VCAM1, NOS3, GSTP1, MMP2, FOS, MAPK14, SELE, TNF, MMP9, RELA, VEGFA, IKBKB, BCL2, AKT1, HMOX1, TP53*
Hsa05020: Calcium signaling pathway	18.42105263	14	1.59326E-07	*CASP9, GSK3B, PPP3CA, CASP3, BAX, CACNA1S, MAPK14, PRKACA, TNF*
Hsa05010: Alzheimer disease	21.05263	16	1.11E-06	*GSK3B, CHRM1, NOS2, CHRNA7, INSR, PTGS2, TNF, RELA, CASP9, IKBKB, BACE2, PPP3CA, CASP8, CASP3, AKT1, CACNA1S*
Hsa05207: Chemical carcinogenesis-receptor activation	17.10526	13	3.14E-07	*CHRNA7, FOS, ADRB2, CYP3A4, RELA, ESR2, VEGFA, AR, CYP1A1, BCL2, AKT1, CACNA1S, PRKACA*
Hsa05210: Colorectal cancer	11.84211	9	8.40E-07	*CASP9, GSK3B, TGFB1, CASP3, BCL2, BAX, AKT1, FOS, TP53*
Hsa04728: Dopaminergic synapse	13.15789	10	2.33E-06	*GSK3B, PPP3CA, GRIA2, AKT1, FOS, DRD1, MAPK14, PRKACA, SLC6A3, DRD4*
Hsa04370: VEGF signaling pathway	10.52632	8	8.03E-07	*CASP9, PPP3CA, NOS3, PLCG2, AKT1, MAPK14, PTGS2, VEGFA*
Hsa01524: Platinum drug resistance	10.52632	8	3.47E-06	*CASP9, CASP8, CASP3, GSTP1, BCL2, BAX, AKT1, TP53*
Hsa04726: Serotonergic synapse	11.84211	9	7.61E-06	*ALOX5, CASP3, HTR2C, CACNA1S, HTR2A, PRKACA, PTGS2, SLC6A4, PTGS1*
Hsa04064: NF-kappa B signaling pathway	10.52632	8	3.63E-05	*IKBKB, VCAM1, PLAU, BCL2, PLCG2, PTGS2, TNF, RELA*
Hsa04931: Insulin resistance	10.52632	8	4.64E-05	*IKBKB, GSK3B, NOS3, INSR, SLC2A2, AKT1, TNF, RELA*
Hsa04211: Longevity regulating pathway	9.210526	7	1.34E-04	*INSR, BAX, AKT1, PPARG, PRKACA, TP53, RELA*
Hsa04218: Cellular senescence	10.52632	8	4.60E-04	*CCNA2, PPP3CA, TGFB1, CDK1, AKT1, MAPK14, TP53, RELA*

### 3.6 Construction of “composition-target” network and “herb-component-target-pathway-disease” network

**Figure 6:**
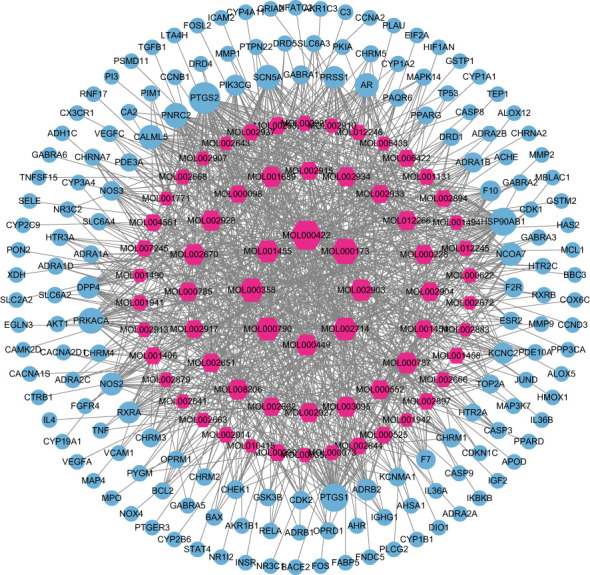
Composition-target network of HLJDD. The red hexagon is the active ingredient of the drug; and the blue circle is the target.

The data of potential active ingredients and potential targets of HLJDD in the treatment of AD were imported into Cytoscape 3.9.1 software to obtain a diagram of the traditional Chinese medicine composition-target network (**Figure 6**). The data showed that kaempferol, wogonin, beta sitosterol, baicalein, acacetin, isosorbine, (S)-cardione, (R)-cardione were the top 8 active ingredients with the degree values of 37, 23, 21, 21, 18, 17, 16 and 15 respectively, which suggested that they may be the main chemical active ingredients of HLJDD for the treatment of AD (**Figure 6**).

**Figure 7:**
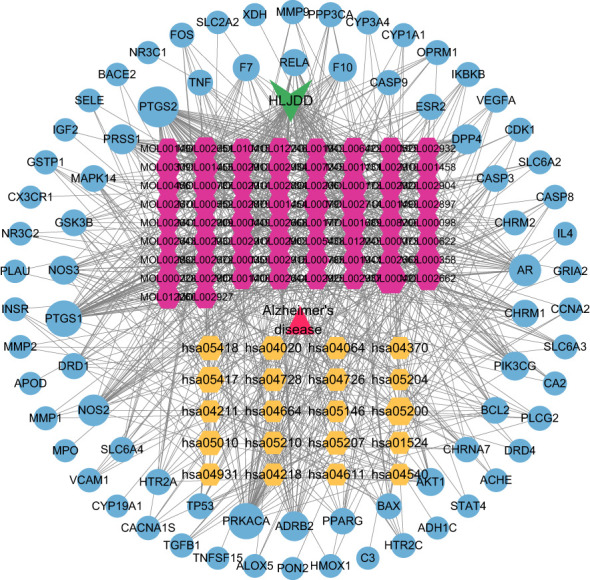
H-C-T-P-D network diagram. Triangle represents disease; V is HLJDD; The yellow hexagon is the KEGG pathway; The red hexagon is the active ingredient of the drug; The blue circle is the target.

The “herb-component-target-pathway-disease” network was constructed using the 66 active ingredients, 76 intersection targets, and 15 KEGG signal pathways of HLJDD in the treatment of AD (**Figure 7**). It showed that multiple active ingredients were related to multiple targets and pathways, and the therapeutic effect of HLJDD may be achieved by working multiple active ingredients in conjunction with multiple targets.

### 3.7 Molecular docking results and analysis

**Figure 8:**
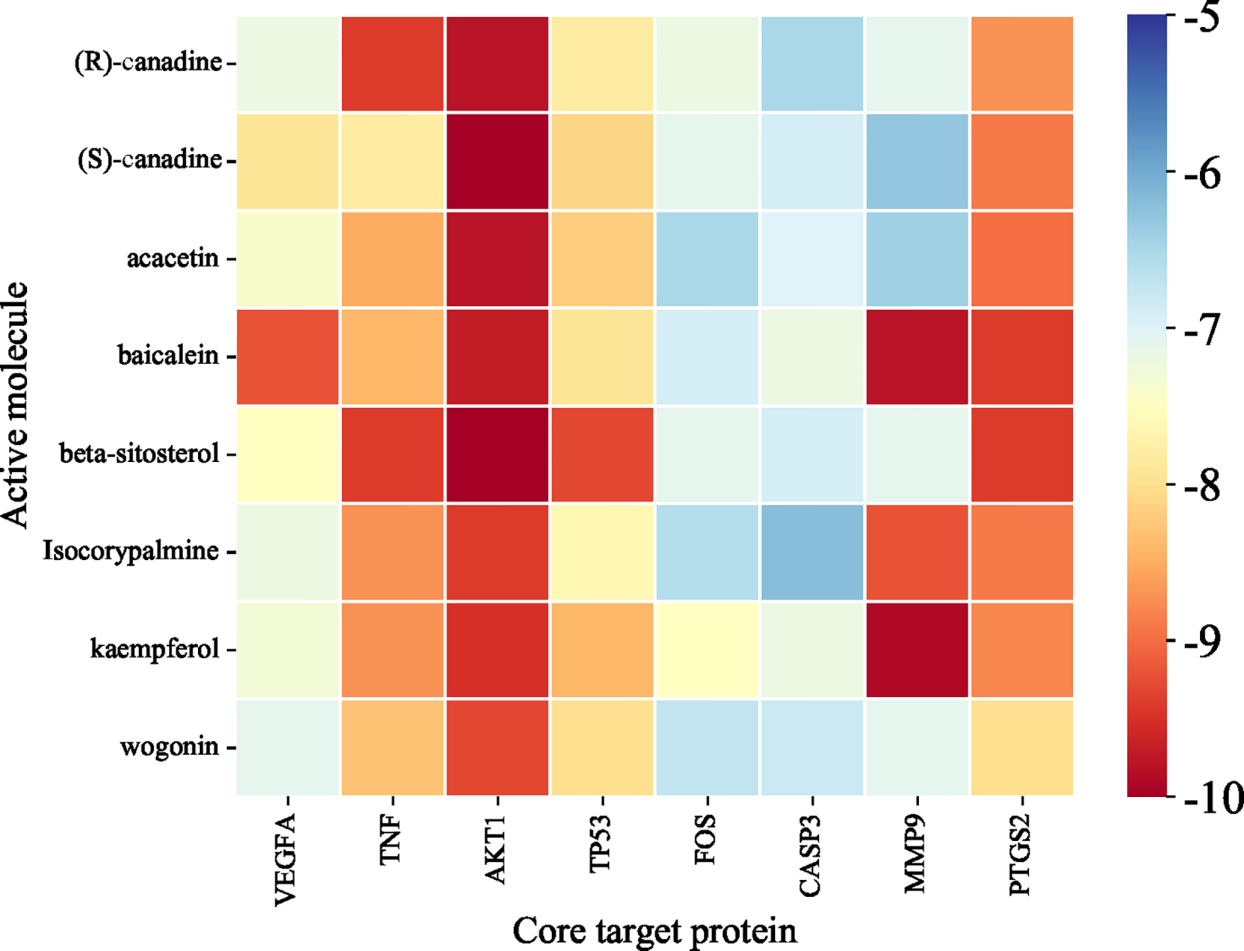
Molecular docking heatmap of chemical compositions to targets.

**Figure 9:**
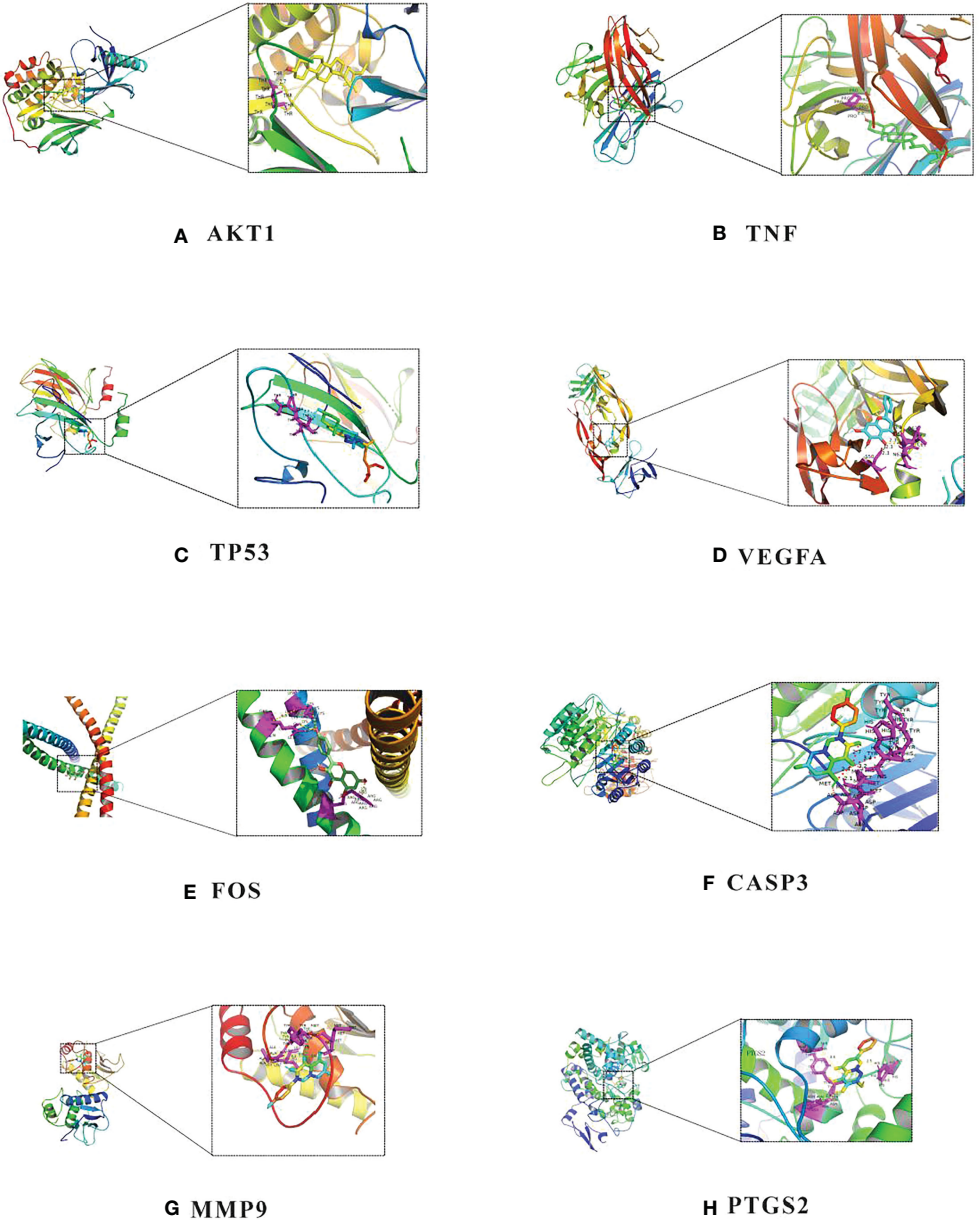
Molecular docking diagram of chemical composition to target: **(A)** AKT1 to beta-sitosterol; **(B)** TNF to beta-sitosterol; **(C)** TP53 to kaempferol; **(D)** VEGFA to baicalein; **(E)** FOS to kaempferol; **(F)** CASP3 to kaempferol; **(G)** MMP9 to kaempferol; **(H)** PTGS2 to baicalein.

According to **Table 2**, the top 8 targets of degree are AKT1, TNF, TP53, VEGFA, FOS, CASP3, MMP9, PTGS2. The docking targets with the 8 active components with the highest degree of kaempferol (degree = 37), wogonin (degree = 23), beta-sitosterol (degree = 21), baicalein (degree = 21), acacetin (degree = 18), isosorbine (degree = 17), (S)-cardione (degree = 16), (R)-cardione (degree = 15) in HLJDD were performed docking. As shown in **Table 4** and **Figure 8** (heatmap was plotted by https://www.bioinformatics.com.cn), The binding energies of the above eight compounds with AKT1, TNF, TP53, VEGFA, FOS, CASP3, MMP9, and PTGS2 were less than -5.0 kcal · mol^-1^, showing good binding ability. The binding of AKT1 to beta-sitosterol, TNF to beta-sitosterol, TP53 to kaempferol, VEGFA to baicalein, FOS to kaempferol, CASP3 to kaempferol, MMP9 to kaempferol, and PTGS2 to baicalein were shown in **Figure 9**.

**Table 4:** Docking results of target proteins and active compounds.

Core target	PDB ID	Binding energy (kcal/mol)								
		kaempferol	wogonin		beta-sitosterol	baicalein	acacetin	isocorypalmine	(S)-canadine	(R)-canadine
AKT1	6s9x	-9.5	-9.3	-11.5		-9.7	-9.8	-9.4	-10	-9.8
TNF	6op0	-8.7	-8.3	-9.4		-8.4	-8.5	-8.7	-7.8	-9.4
TP53	5mf7	-8.4	-8	-9.3		-7.9	-8.2	-7.6	-8.1	-7.8
VEGFA	4qaf	-7.3	-7.1	-7.5		-9.2	-7.4	-7.2	-7.9	-7.2
FOS	1fos	-7.5	-6.7	-7.1		-6.9	-6.5	-6.6	-7.1	-7.2
PTGS2	5ikt	-8.8	-8	-9.4		-9.4	-9	-8.9	-8.9	-8.7
MMP9	1gkd	-9.9	-7.1	-7.1		-9.8	-6.4	-9.2	-6.3	-7.1
CASP3	3dej	-8.8	-8	-9.4		-9.4	-9	-8.9	-8.9	-8.7

## 4 Discussion

HLJDD, which is the representative medicine of heat-clearing and detoxification of traditional Chinese medicine, has been reported to show significant anti-inflammatory and neuroprotective effects in the neurodegenerative diseases. In our study, bioinformatics analysis revealed that kaempferol, wogonin, beta-sitosterol, baicalein, acacetin, isocorypalmine, (S)-canadine, (R)-canadine may be potential candidate agents of HLJDD; AKT1, TNF, TP53, VEGFA, FOS, PTGS2, MMP9 and CASP3 could become potential therapeutic targets in the treatment of AD.

According to the GO and KEGG pathway enrichment analysis results, we speculated that the mechanism of HLJDD in the treatment of AD may be mainly related to VEGF, NF-κB, Ca^2+^ signaling pathways. Recent studies have shown that VEGF, NF-κB, and Ca^2+^ are all involved in the process of inflammation, which is closely associated to AD (Kirk and Karlik, 2003; Thawkar and Kaur, 2019; Cheng et al., 2021). AKT1 is an important member of the AKT (protein kinase B, PKB) family that regulates cell proliferation and growth, and its activation is mainly dependent on the PI3K signaling pathway (Kumar and Bansal, 2022). PI3K/AKT signal transduction pathway is involved in a variety of cellular and biological processes *in vivo*. Previous study also found that activation of the PI3K/AKT/FoxO3a pathway, can play a role in the reduction of inflammatory response in AD mice and restoring the therapeutic effect of cognitive impairment (Wang et al., 2020). Tumor necrosis factor (TNF) is a small molecule protein mainly secreted by macrophages. TNF is involved in a variety of cellular processes, including activating NF-κB signaling pathway, promoting cell death and regulating immune function (Webster and Vucic, 2020). Studies have shown that TNF-mediated neuroinflammation was associated with necroptosis of hippocampal neurons in AD. Therefore, TNF-α-targeted therapy was a biologically feasible approach to prevent or attenuate AD (Decourt et al., 2017; Torres-Acosta et al., 2020; Jayaraman et al., 2021). Vascular endothelial growth factor (VEGF), a highly specific vascular endothelial growth factor, can promote vascular permeability, angiogenesis, blood production, and neural development (Robinson and Stringer, 2001). Studies have shown that VEGF can improve the spatial learning and memory ability of AD mice and reduce the level of Aβ. In addition, VEGF protected SH-SY5Y cells against Aβ_25-35_-induced neurotoxicity by improving mitochondrial function and numbers, increasing neuronal activity and reducing intracellular ROS production (Liu et al., 2021). Moreover, the high concentration of VEGF-A in cerebrospinal fluid was related to the slower cognitive decline in patients with AD risk (Hohman et al., 2015).

It was reported that kaempferol, a potentially active ingredients in HLJDD, significantly protected neurons and SH-SY5Y cells from rotenone-induced injury by reducing protease lysis, nuclear apoptosis, the level of oxidative stress and mitochondrial hydroxyl compounds (Filomeni et al., 2012). In addition, kaempferol attenuated STZ-induced memory impairment in OVX rats by increasing hippocampal endogenous superoxide dismutase and glutathione levels and reducing neuroinflammation (Kouhestani et al., 2018). Ji et al. found that wogonin and baicalein effectively relieved Aβ_25-35_-stimulated PC12 cell apoptosis and inflammation (Ji et al., 2020). Moreover, wogonin and baicalein inhibited apoptosis and production of inflammatory factors TNF-α, NO and PGE2 (Ji et al., 2020). β-sitosterol improved memory, learning impairment and reduced Aβ deposition in APP/PS1 mice through helping reverse the loss of dendritic spines in APP/PS1 mice, and the decrease of miniature excitatory postsynaptic current frequency in hippocampal neurons (Ye et al., 2020). In addition, β-sitosterol inhibited LPS-induced inflammatory response in BV-2 cells by inhibiting the activation of ERK, p38 and NF-κB pathways (Sun et al., 2020).

Recently, emerging studies have confirmed that neuroinflammation is a key factor in the neurodegeneration of AD (Tejera et al., 2019). However, it should be noted that neuroinflammation was not limited to the contributions of relevant factors residing in the brain, as perturbations in microbial diversity were associated with the spread of neuroinflammation in preclinical models of AD. Gut microbes could directly affect the immune system by activating the vagus nerve (Borovikova et al., 2000; Bravo et al., 2012), which in turn occurred bidirectional communication with the central nervous system, thereby linking them to the cognitive and emotional centers of brain (Grenham et al., 2011; Mayer and Tillisch, 2011; Rogers et al., 2016). It has been proven that *Lactobacillus* and *Bifidobacterium* produced GABA; *Escherichia coli*, *Bacillus* and *yeast* produced norepinephrine; *Candida*, *streptococcus*, *Escherichia coli* and *enterococci* produced 5-HT (Dinan et al., 2013); *Lactobacillus* regulated dopaminergic pathways to improve tic-like behavior (Liao et al., 2019); *Bifidobacteria* significantly reduced plasma C-reactive protein, TNFα and IL-6 levels to exert immunomodulatory effects (Groeger et al., 2013). Wang et al. reported that neuroinflammation caused by gut dysbacteriosis promoted the progression of AD. GV-971, a sodium oligomannate, showed stable and sustained cognition improvement in a phase 3 clinical trial in China. It suppressed gut dysbacteriosis and related phenylalanine/isoleucine accumulation, harnessed neuroinflammation, and reversed cognitive dysfunction (Wang et al., 2019b). Gu et al. found that gut dysbacteriosis and lipid metabolism were highly correlated with AD-like neuroinflammation. HLJDD suppressed gut dysbacteriosis and Aβ accumulation, improved neuroinflammation and reversed cognitive dysfunction (Gu et al., 2021).

In addition to the gut microbiota, oral microorganisms, skin microorganisms and pulmonary pathogen also directly or indirectly affected the central nervous system. For example, Weaver reported that oral microorganisms that entered the CNS through the blood-brain barrier (BBB) triggered immune responses in the body and increased the production of Aβ, thus promoted the occurrence of AD (Weaver, 2020). Zeng et al. found that the mice infected with *P. gingivalis* had reduced BBB integrity and increased Aβ content flowing into the brain from the periphery, thereby promoted the occurrence of AD (Zeng et al., 2021). In the context of the gut-brain-skin axis, Wang et al. explored the inflammatory and immune mechanisms of psoriasis and depression (Wang et al., 2021). Specifically, psoriasis and depression can cause gut dysbiosis through the gut-skin axis and the gut-brain axis. In turn, the disorder of gut microbiome can aggravate the inflammatory response in psoriasis and depression. In general, disorders of the gut-brain-skin axis can lead to a vicious cycle of psoriasis and depression. Moreover, Balin et al. demonstrated that *C. pneumoniae* infected the brain. The authors correlated the inflammatory response stimulated by *C. pneumoniae* with the neuroinflammation of late-onset Alzheimer’s disease, and established an animal model of *C. pneumoniae* infection-triggered *in vivo* neuropathology consistent with AD pathology (Balin et al., 2008).

Therefore, we hypothesize that HLJDD played a therapeutic role in AD by regulating the homeostasis of microbial flora and inhibiting the neuroinflammatory response (**Figure 1**). Based on the multidisciplinary strategy, our study provided evidence for the therapeutic effect of HLJDD in AD, and also provided a comprehensive method for finding active compounds, core target genes and potential mechanisms in traditional Chinese medicine, and provided a new theoretical basis for further experimental research and clinical application.

## Data availability statement

The datasets presented in this study can be found in online repositories. The names of the repository/repositories and accession number(s) can be found in the article/Supplementary Material.

## Author contributions

RZ, JG, and PJ contributed to the conception of the study. RZ, SS, QZ, and PJ contributed significantly to analysis and manuscript preparation. SY and TG helped perform the analysis with constructive discussions. All authors contributed to the article and approved the submitted version.
